# Endoleak after use of the fenestrated frozen elephant trunk technique to treat acute type A aortic dissection

**DOI:** 10.1093/jscr/rjae291

**Published:** 2024-05-05

**Authors:** Takeyuki Kanemura, Yoshinori Nakahara, Toshiya Fukushima, Shuhei Kawamoto, Kazuki Morooka, Motoharu Shimozawa

**Affiliations:** Department of Cardiovascular Surgery, IMS Katsushika Heart Center, 3-30-1 Horikiri, Katsushika Ward, Tokyo 124-0006, Japan; Department of Cardiovascular Surgery, IMS Katsushika Heart Center, 3-30-1 Horikiri, Katsushika Ward, Tokyo 124-0006, Japan; Department of Cardiovascular Surgery, IMS Katsushika Heart Center, 3-30-1 Horikiri, Katsushika Ward, Tokyo 124-0006, Japan; Department of Cardiovascular Surgery, IMS Katsushika Heart Center, 3-30-1 Horikiri, Katsushika Ward, Tokyo 124-0006, Japan; Department of Cardiovascular Surgery, IMS Katsushika Heart Center, 3-30-1 Horikiri, Katsushika Ward, Tokyo 124-0006, Japan; Department of Cardiovascular Surgery, IMS Katsushika Heart Center, 3-30-1 Horikiri, Katsushika Ward, Tokyo 124-0006, Japan

**Keywords:** fenestrated FET, frozen elephant trunk, acute type A dissection

## Abstract

Several studies have indicated that the fenestrated frozen elephant trunk (FET) technique enhances early outcomes in cases of acute aortic dissection, although long-term outcomes remain unclear. A case involving a 62-year-old male who experienced endoleak from a fenestration site following total arch replacement using the fenestrated FET technique for a DeBakey type I aortic dissection is reported. The patient underwent successful reoperation involving total arch replacement and reinsertion of the FET. Postoperatively, there was an absence of endoleak from the fenestration, and a noteworthy reduction in the diameter of the aortic arch was observed. It is imperative to recognize that endoleak from a fenestration poses a risk for prompt aortic expansion, thus necessitating vigilant postoperative monitoring. Furthermore, when adopting fenestrated FET, it is crucial to ensure firm fixation around the fenestration to prevent endoleak.

## Introduction

The frozen elephant trunk (FET) is one of several surgical techniques for total arch replacement in aortic dissection, and is recommended for patients with primary entry in the distal aortic arch or in the proximal half of the descending thoracic aorta to treat associated malperfusion syndrome or to avoid its postoperative development [1]. Favorable outcomes have been reported using the fenestrated FET technique, in which the stent graft is fenestrated, and the cervical vessels are perfused through the fenestration [2]. This method has the advantages of more proximal distal anastomosis, technical simplicity and positive remodeling of the descending aorta. However, fenestration-related endoleak remains a potential complication. We report a case of endoleak 6 months after total arch replacement using the fenestrated FET technique in a patient with DeBakey type I aortic dissection (DTAAD), which necessitated reoperation.

## Case report

**Figure 1:**
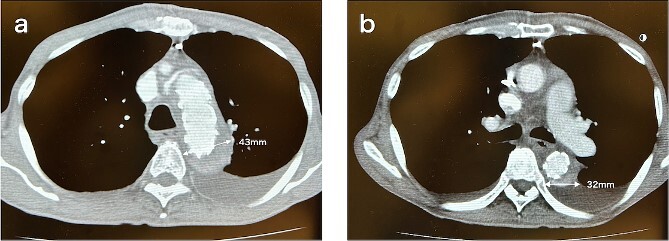
CT 5 days after the initial surgery revealed no endoleak and good remodeling of the descending aorta at the levels of the aortic arch (a) and the carina of the trachea (b).

**Figure 2:**
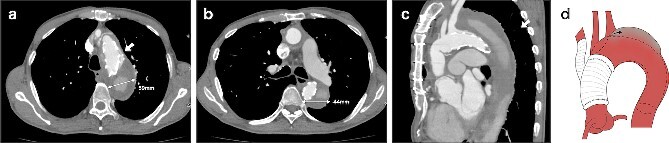
CT 6 months after the initial surgery reveals an endoleak from the fenestration (indicated by the white arrow), antegrade flow in the false lumen, and an enlarged aorta at the levels of the aortic arch (a) and the carina of the trachea (b). Sagittal image (c). An illustrative diagram (d) shows the schema of endoleak from fenestration (indicated by the black arrow).

**Figure 3:**
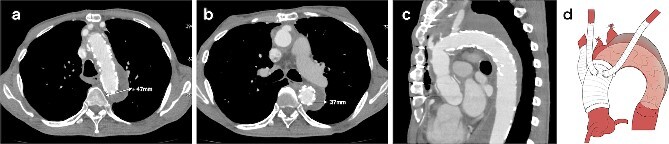
CT 3 months after redo surgery reveals no endoleak and no flow in the false lumen, and remodeling of the aorta at the levels of the aortic arch (a) and the carina of the trachea (b). Sagittal image (c). An illustrative diagram (d) shows the postoperative schema.

A 62-year-old male presented at the previous hospital with left hemiplegia. Computed tomography (CT) detected an aortic dissection with malperfusion of the right brachiocephalic artery, necessitating a referral to our institution. Preoperative CT showed the primary entry tear in the distal aortic arch and a patent false lumen. A total arch replacement was successfully performed using the fenestrated FET technique. The surgical procedure involved median sternotomy, establishment of extracorporeal circulation with right atrial drainage and perfusion via right axillary artery, circulatory arrest at 28°C and selective cerebral perfusion to perfuse all cervical vessels. Myocardial protection was provided by retrograde cardioplegia. Following resection of the aorta at zone 1, FET was inserted and deployed into the aorta, perfusing blood from the femoral artery. Fenestration of the graft at the ostium of the second and third cervical branches was performed. A U-shaped graft was placed around cervical branches, and three 4-0 polypropylene U-shaped sutures were roughly placed to fix the open stent graft, leaving some gaps between the sutures. Subsequent procedures included anastomosis of a four-branch graft to the distal aorta, reconstruction of the brachiocephalic artery and proximal anastomosis. Postoperative contrast-enhanced CT on day 5 showed no endoleak and favorable remodeling of the descending aorta (Fig. 1). However, after 6 months, follow-up CT detected a fenestration-related endoleak and an enlarged aortic arch (Fig. 2), prompting reoperation. The reoperation involved median re-sternotomy, establishment of extracorporeal circulation with right femoral vein drainage and perfusion via an 8-mm graft anastomosed to the left axillary artery, circulatory arrest at 28°C, and selective cerebral perfusion to perfuse all cervical vessels. Upon opening the artificial graft, it was observed that the stent graft had some gap between fixed U-sutures and had become detached from the aorta, revealing a small entry adjacent to it, which communicated with the false lumen. The left subclavian artery (LSCA) was ligated, and the left common carotid artery was transected at its origin and sutured. An open stent was inserted distally, followed by placement of a felt strip and a 4-0 running suture. Subsequent procedures included anastomosis of a four-branch graft to the distal aorta, reconstruction of the three cervical branches and proximal anastomosis. The surgery was completed without complications. Postoperative contrast-enhanced CT on day 5 revealed resolution of the endoleak with no blood flow into the false lumen of the arch. Follow-up CT 3 months after discharge revealed favorable remodeling of the aortic arch (Fig. 3).

## Discussion

We encountered a case of fenestration-related endoleak after total arch replacement using a fenestrated FET in a patient with DTAAD. The fenestrated FET has been reported to yield favorable results, including improved early surgical outcomes [2]. However, there are complications, including fenestration-related endoleak, for which the late outcome remains unknown. In the case described herein, the distal aorta was promptly enlarged, which clarified that endoleak from the fenestration could cause a late aortic event. Endoleaks identified using follow-up CT should be carefully monitored.

Fixation is a key to prevent fenestration-related endoleaks. A previous study reported good results without fixation [3], while Okamura *et al.* [2], in contrast, recommended the use of fixation. We have performed 26 fenestrated FET procedures and encountered three endoleaks (11%). For this reason, we also suggest that fixation should be used for fenestrated FET. There are two methods for fixation. First, the LSCA is encircled with a U-shaped piece of artificial graft, and 4-0 polypropylene running sutures are performed around the fenestration site [2]. Second, 4-0 polypropylene U-shaped sutures are placed around the LSCA with a U-shaped piece of an artificial graft. We had predominantly favored the latter method due to the convenience of U-sutures for deeper needling into the aorta behind the LSCA. After the instances where endoleaks occurred due to gaps between U sutures, we implemented overlapping U-shaped stitches to mitigate this issue. Conversely, the use of continuous sutures, which eliminates gaps between stitches, may potentially reduce the likelihood of endoleaks.

In this case, we were able to safely reoperate for fenestration-related endoleak by reconstructing the cervical vessels and reinserting of the FET. Although we chose to the re-sternotomy due to the young age of the patient, endovascular repair is one option. A previous case report described successful coil embolization of the false lumen in the aortic arch [4]. Thoracic endovascular aortic repair (i.e. ‘TEVAR’) could also be considered. Some studies suggest that TEVAR is an effective and safe treatment for distal stent-graft induced new entry after FET procedure [5, 6]. The fenestration-related endoleak, occurring near the origin of the neck vessel, necessitates debranching. Moreover, it is critical to position the reconstructed brachiocephalic artery or the right common carotid artery origin more proximal to secure an adequate proximal landing zone, a crucial consideration for fenestrated FET procedures. In case of two fenestrations in the initial surgery, two debranchings are required in additional surgery for fenestration-related endoleak. On the other hand, one fenestration requires only one debranching, thus making reoperation easier. For patients at a high risk of re-sternotomy, such as the elderly or those with impaired respiratory function, one fenestration would be the better choice for the initial fenestrated FET for a DTAAD.

In conclusion, our experience highlights the importance of ensuring secure fixation around fenestrations to prevent endoleak in fenestrated FET procedures for aortic dissection. Reoperation successfully addressed the complication, emphasizing the necessity of meticulous follow-up for fenestration-related endoleak.
